# Nonsurgical periodontal therapy remodels oral microbiome-metabolome networks and associates with glycemic and inflammatory improvements in type 2 diabetes mellitus with periodontitis: a 6-month longitudinal study

**DOI:** 10.1080/20002297.2026.2660482

**Published:** 2026-04-17

**Authors:** Jing Diao, Hejia Li, Shuguo Zheng, Jiahui Niu, Chao Yuan

**Affiliations:** a Department of Preventive Dentistry, Peking University School and Hospital of Stomatology & National Center for Stomatology & National Clinical Research Center for Oral Diseases & National Engineering Research Center of Oral Biomaterials and Digital Medical Devices & Beijing Key Laboratory of Digital Stomatology, Beijing, People's Republic of China; b Third Clinical Division, Peking University School and Hospital of Stomatology & National Center for Stomatology & National Clinical Research Center for Oral Diseases & National Engineering Research Center of Oral Biomaterials and Digital Medical Devices & Beijing Key Laboratory of Digital Stomatology, Beijing, People's Republic of China; c Department of Stomatology, the Second Hospital of Shijiazhuang City, Hebei, People's Republic of China

**Keywords:** Type 2 diabetes mellitus, periodontitis, non-surgical periodontal therapy, saliva, gingival crevicular fluid, microbiome, metabolome

## Abstract

**Background:**

Periodontitis and type 2 diabetes mellitus (T2DM) have a bidirectional relationship, yet how nonsurgical periodontal therapy (NSPT) affects oral microbiome-metabolome interactions in this comorbidity remains unclear.

**Objective:**

To longitudinally characterize oral microbiome and metabolome shifts following NSPT in patients with T2DM and periodontitis, and to relate these shifts to periodontal and systemic outcomes.

**Design:**

A total of 42 participants completed the 6-month follow-up. At baseline, they were randomly assigned to NSPT (supragingival scaling plus scaling and root planning, *n* = 24) or supragingival scaling alone (*n* = 18). Periodontal parameters, glycated hemoglobin (HbA1c), C-reactive protein (CRP), and glucose/lipid markers were measured at baseline, at 3 and 6 months. Oral samples were collected at each visit for microbiome profiling and metabolomics. Microbe-metabolite-clinical features and associations were explored using correlation analyses and pathway annotation.

**Results:**

NSPT improved periodontal inflammation and was accompanied by favorable changes in systemic inflammation and glucose/lipid indices; HbA1c reduction was more pronounced in those with poorer baseline glycemic control. Both groups showed temporal variability in microbiome and metabolome profiles, but the taxa/metabolites that changed differed between groups. Changes in bleeding index correlated positively with 13-eicosenoic acid, xylose, *Rothia aeria*, and *Alloprevotella tannerae*, whereas changes in CRP correlated positively with sorbitol, galactitol, *Prevotella nigrescens,* and *Treponema denticola*. Selected microbe-metabolite pairs mapped to glutathione and purine metabolism.

**Conclusion:**

NSPT reshapes oral microbe-metabolite networks in T2DM with periodontitis, implicating redox-related pathways that may link periodontal therapy to systemic inflammation and glycemic control.

## Introduction

Periodontitis is a chronic infectious disease driven by periodontal pathogens and ultimately leads to local periodontal inflammation, loss of periodontal attachment, and alveolar bone resorption [1]. Evidence suggests that individuals with more severe periodontal inflammation have poorer glycemic control than periodontally healthy individuals, with HbA1c control deteriorating over time [2–4], and are at higher risk of developing incident type 2 diabetes mellitus (T2DM) and gestational diabetes [5]. Moreover, diabetic patients with periodontitis experience more severe diabetic complications than those with little or no periodontitis [6]. Regarding the impact of periodontitis on diabetes, some studies have proposed that inflammatory mediators derived from periodontal lesions enter the circulation and induce systemic inflammation [7], whereas others suggest that periodontal pathogens exacerbate insulin resistance through multiple pathways, ultimately contributing to suboptimal glycemic control in patients with diabetes [4,8,9].

Nonsurgical periodontal therapy (NSPT), comprising supragingival scaling, subgingival scaling and root planning, aims to remove local aetiologic factors, thereby resolving inflammation and promoting the regain of periodontal attachment. Previous studies have shown that NSPT can improve glycemic control in patients with diabetes [10–14] and reduce circulating inflammatory mediators [7,15]. By disrupting the oral plaque biofilm, such therapy may decrease the inflammatory burden and systemic inflammation, which has been proposed as a mechanism underlying its glucose-lowering effect [16]. To investigate the effects of NSPT on the oral microbiota, Shi et al. [17] used high-throughput sequencing to evaluate changes in the subgingival microbial community in periodontitis patients with and without T2DM before and after treatment. They observed decreased levels of *Prevotella intermedia, Porphyromonas gingivalis*, and *Tannerella forsythia*; however, patients with poor glycemic control exhibited a smaller shift in microbial community structure and a weaker tendency to revert toward a health-associated state. Similarly, Silva et al. [18] found that periodontal pathogens decreased after NSPT in both groups, but the magnitude of reduction was less pronounced in the T2DM group than in normoglycemic individuals. Collectively, these findings suggest that although NSPT reduces the abundance of periodontal pathogens, its microbiome-modulating effects appear attenuated in patients with T2DM, particularly among those with suboptimal glycemic control.

Metabolites are terminal products of gene expression and integral components of regulatory networks. Evidence indicates that a key mechanism by which the oral microbiota influences host physiology is through the production of small-molecule compounds that enter host tissues and circulation, thereby participating in metabolic processes at both local and systemic levels [19]. Current oral metabolomics research has largely focused on systemically healthy individuals with periodontitis [20–23]. Integrative analyses combining oral microbiomics and oral metabolomics enable systematic investigation of the interplay between oral microbial communities and metabolite profiles [24]. Several studies [25,26] have shown that metabolite profiling of saliva and dental plaque can uncover candidate metabolic biomarkers and pathways associated with dental caries and periodontitis, thereby creating opportunities to validate early diagnostic models and to identify novel targets for the prevention and treatment of oral diseases and T2DM. However, how NSPT reshapes the oral microbiome and metabolome in patients with T2DM and periodontitis, how these two omics layers are interrelated, and whether such changes are associated with glycemic control remain to be elucidated.

Therefore, this study investigated therapy-associated changes in the oral microbiome and metabolome in patients with T2DM and periodontitis before and after NSPT. By integrating these omics data with periodontal parameters and systemic indices of glyco-lipid metabolism and inflammation, we sought to delineate potential links among these measures, identify implicated metabolic pathways, and provide a basis for mechanistic studies on how periodontitis influences diabetes. This work may advance understanding of the microbiome-metabolome-inflammation axis and offer new insights for personalised periodontal therapy and diabetes management, as well as potential targets for future prevention strategies.

## Materials and methods

### Ethics and consent

The protocol received approval from the Peking University Biomedical Ethics Committee (approval ID: PKUSSIRB-201944042), and the study was prospectively registered in the Chinese Clinical Trial Registry (ChiCTR1900023582). Written informed consent was obtained from all participants before any procedures. The same governance and consent processes were applied at each longitudinal visit.

### Study design and participants

Participants were enroled between April and October 2019. At baseline, 50 participants were enroled and randomly allocated in a 1:1 ratio to the intervention group (group A) or control group (group B) using a random-number table. Baseline periodontal examinations were performed, and whole blood, unstimulated whole saliva, and gingival crevicular fluid (GCF) were collected from all participants. Standardised oral hygiene instruction (OHI) was provided to every participant.

**Figure 1.**
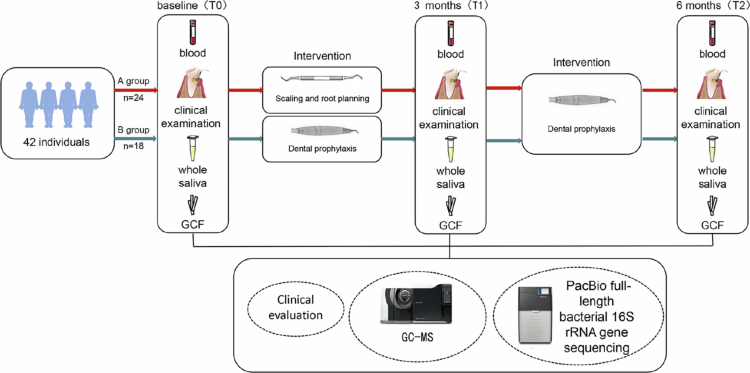
The flowchart of this study showing the enrolment and sample collection, as well as the classification of participants for subsequent microbiota and metabolite analyses.

The intervention consisted of supragingival scaling, subgingival scaling, and root planning in the intervention arm, whereas the control arm received supragingival scaling only. At the 3-month visit, all participants underwent periodontal re-examination; whole blood, unstimulated saliva, and GCF were recollected; and OHI with maintenance supragingival debridement was provided. At the 6-month visit, periodontal examinations and collection of whole blood, unstimulated saliva, and GCF were repeated for all participants. Finally, a total of 42 participants completed the 6-month follow-up and full biospecimen schedule (intervention *n* = 24; control *n* = 18). Upon study completion, compensatory NSPT was offered to the control arm. The study workflow is summarised in Figure 1.

Eligibility required: age 50-75 years; ≥ 15 natural teeth (third molars excluded); a documented diagnosis of type 2 diabetes mellitus (T2DM) for ≥ 1 year according to the latest American Diabetes Association criteria; and no modification in antidiabetic regimen (dose or formulation) within the preceding 3 months; non-smoking (i.e. no smoking behaviour in the past year, and smoked fewer than 100 cigarettes prior to enrollment); has signed the informed consent form and is able to comply with subsequent study procedures. Exclusions comprised systemic diseases other than T2DM (e.g. recent major cardiovascular events, stroke, renal failure, hepatic dysfunction), advanced diabetic complications, and active infections (e.g. periapical abscess).

Periodontal examinations were conducted by two calibrated periodontists using a manual probe (PCPUNC 15; Hu-Friedy), recording probing depth (PD), clinical attachment loss (CAL), and bleeding index (BI) at six sites per tooth. Group matching by age and sex was attempted at baseline. The periodontal clinical examination was conducted after saliva and GCF sampling.

### Sample collection

Participants abstained from food, beverages, and oral hygiene measures for at least 2 h before sampling, rinsed with water, and rested for 10 min to standardise conditions. Unstimulated whole saliva (2 mL) was collected between 08:00 and 09:00, transported on ice, and centrifuged (10,000 × g, 10 min, 4 °C). Supernatants were aliquoted (200 µL) and, together with corresponding pellets, stored at −80 °C. GCF was sampled at the same sextet of teeth (11, 31, 16, 26, 36, 46) for all visits. After cotton roll isolation, gentle removal of supragingival plaque, and air-drying, paper strips were inserted 1–2 mm into the sulcus/pocket for 30 s. Strips from each participant were pooled into coded tubes containing 350 µL sterile PBS, vortexed (2 min), shaken (40 min, 4 °C), and centrifuged (10,000 × g, 10 min, 4 °C). Supernatants were discarded; pellets were stored at −80 °C pending DNA extraction. Strips contaminated with blood were excluded.

Blood samples were collected and analysed. Prior to sampling, participants were instructed to avoid fatty foods within 24 h and to abstain from food and water for at least 8 h. Fasting venous blood was collected between 08:00 and 09:00, followed by serum biochemical measurements. The assessed biochemical parameters included glycated haemoglobin (HbA1c), fasting plasma glucose (FPG), total cholesterol (CHOL), triglycerides (TG), high-density lipoprotein (HDL), low-density lipoprotein (LDL), and C-reactive protein (CRP).

The above procedures were identically applied at each longitudinal visit.

### Microbial DNA extraction and full-length 16S rRNA sequencing

DNA from saliva and GCF pellets was isolated using the QIAamp DNA Mini Kit (Qiagen) per manufacturer instructions. DNA concentration and purity were assessed by NanoDrop 8000; integrity was checked by 1.2% agarose electrophoresis with a buffer-only negative control. DNA was stored at −80 °C. Nearly full-length bacterial 16S rRNA genes were amplified using primers 27F (5′-AGAGTTTGATCMTGGCTCAG-3′) and 1492 R (5′-ACCTTGTTACGACTT-3′). Cycling conditions were: 98 °C for 2 min; 25–30 cycles of 98 °C for 15 s, 55 °C for 30 s, 72 °C for 30 s; final extension 72 °C for 5 min; hold at 10 °C. Amplicons were sequenced on the PacBio Sequel platform (Shanghai Personal Biotechnology, China). The same extraction, amplification, and sequencing steps were used for all time points.

Raw paired-end reads were processed using QIIME2 (v2019.4) with cutadapt for primer trimming and DADA2 for denoising, paired-end merging, and chimera removal, generating amplicon sequence variants (ASVs). Feature tables were created from these ASVs, and singleton ASVs (with counts of 1 across all samples) were removed. To ensure consistency in diversity analyses, ASV feature tables were rarefied to a uniform sequencing depth (95% of the minimum sequencing depth across samples).

### Preparation of salivary supernatant and GC-MS metabolomics

For broad-coverage targeted analysis, 200 µL of acetonitrile/methanol (1:1, v/v) containing 10 µg/mL tridecylic acid (internal standard) was combined with 200 µL salivary supernatant, vortexed (30 s), and centrifuged (14,000 rpm, 10 min, 4 °C). Approximately 350 µL supernatant was dried in a centrifugal concentrator; 50 µL of methoxylamine hydrochloride (15 mg/mL in pyridine) was added and incubated (37 °C, 90 min). Subsequently, 40 µL MSTFA was added for derivatization (37 °C, 60 min), followed by centrifugation (14,000 rpm, 10 min, 4 °C); supernatants were injected for GC–MS. Derivatives were separated on an Agilent 7890A–5975C GC–MS with an HP-5MS column (30 m × 0.25 mm × 0.25 µm). Injector was 260 °C; helium carrier gas at 1 mL/min; splitless 1 µL injections. Oven: 60 °C initial; ramp 8 °C/min to 310 °C; hold 6 min. MS: quadrupole 150 °C; ion source 230 °C; electron impact 69–70 eV; full scan m/z 50–600. Samples were run in randomised order to reduce instrumental drift. Quality-control (QC) samples were prepared by pooling equal volumes of all extracts; one QC was analysed after every 10 study samples to monitor stability and repeatability. All salivary supernatant samples collected at baseline, 3 months, and 6 months were derivatized and analysed in a single batch using broad-coverage targeted GC-MS. All analytical conditions were reproduced for each longitudinal batch.

For SCFA extraction, 200 µL saliva was mixed with 200 µL diethyl ether containing deuterobutyric acid (0.5 µL/mL), vortexed (1 min), and centrifuged (12,000 rpm, 20 min, 4 °C); the organic phase was transferred to autosampler vials. Analyses were performed on an Agilent 7890A-5975C GC-MS using an FFAP capillary column (30 m × 0.25 mm × 0.25 µm). Injection volume was 1 µL with a 5:1 split. Oven programme: 100 °C for 1 min; ramp 5 °C/min to 160 °C; ramp 40 °C/min to 240 °C; hold 240 °C for 10 min. Helium flow was 1 mL/min. Temperatures for inlet, transfer line, and ion source were 250 °C, 270 °C, and 230 °C, respectively. Full-scan EI at 70 eV (m/z 20–350) was used. Peak areas were used for quantification. Raw files were processed in MassHunter (Agilent) for peak integration, calibration, and concentration calculation; measurements were performed at Metabo-Profile Biotechnology (Shanghai, China). The same pipeline was used for all visits.

### Data processing and statistical analysis

Data entry and verification were performed using double data entry to ensure accuracy. Statistical analyses of demographic characteristics and clinical parameters were conducted in SPSS version 27.0 (IBM, Armonk, NY, USA). For continuous variables, normality was assessed using the Shapiro-Wilk test. Normally distributed data are presented as mean ± standard deviation; non-normally distributed data are summarised as median (first quartile-third quartile). Between-group comparisons of continuous variables were carried out with the independent-samples t test when assumptions of normality and homoscedasticity were met; otherwise, the Mann-Whitney U test was used. Categorical variables are reported as counts (percentages), and between-group differences in unordered categories (e.g. sex) were evaluated with the chi-square test.

Sequencing data were processed primarily using R (version 3.5.0) and the QIIME2 platform. Differences in the relative abundance of bacterial taxa between groups were assessed with Welch’s t test in STAMP (version 2.1.3). Volcano plots and partial least-squares discriminant analysis (PLS-DA) were generated in MetaboAnalyst (version 6.0, https://dev.metaboanalyst.ca/). The functional enrichment analysis of metabolites was derived with reference to the Kyoto Encyclopaedia of Genes and Genomes (KEGG). Multiple testing correction was performed using the Benjamini-Hochberg false discovery rate (FDR) procedure. Statistical significance was set at two-sided *P* < 0.05.

## Results

### Demographic and clinical characteristics

#### Baseline characteristics of the intervention and control groups

A total of 42 participants completed the 6-month follow-up, including 24 patients in the intervention group (mean age, 68.42 ± 5.69 years) and 18 patients in the control group (mean age, 65.06 ± 5.68 years). At baseline, the intervention group exhibited significantly higher BI, HDL, and CRP levels than the control group (*P *= 0.041, *P *= 0.039, and *P *= 0.032, respectively). Detailed data are presented in Supplementary Table 1.

#### ANCOVA analyses adjusting for baseline values

Given the baseline imbalance between groups, baseline-adjusted analyses were performed using ANCOVA. After adjustment, the intervention group showed significantly lower bleeding index at 3 months (*P *< 0.001) and a borderline significant difference at 6 months (*P *= 0.050) compared with the control group. A significant group effect was also observed for CRP at 3 months after baseline adjustment (*P *= 0.017), whereas no significant adjusted group differences were detected for HDL at either follow-up time point. Detailed ANCOVA results are provided in the Supplementary Table 2.

#### Clinical parameters at the 3- and 6-month follow-up after nonsurgical periodontal therapy

At the 3-month follow-up, the intervention group showed greater improvements in periodontal parameters than the control group, with significantly lower PD and BI and larger reductions in PD, BI, and CAL from baseline (all *P *< 0.05). No significant between-group differences were observed for glyco-lipid metabolic and inflammatory markers at this time point; however, the changes in FPG, CHOL, and CRP from baseline to 3 months differed significantly between groups, showing opposite trends in the intervention (decreased) and control (increased) groups (Supplementary Tables 3 and 4).

At the 6-month follow-up, periodontal improvements were maintained in both groups, with the intervention group demonstrating significantly lower PD and greater reductions in PD and BI compared with the control group (all *P *< 0.05). For metabolic and inflammatory markers, no significant between-group differences were detected at 6 months, although the reduction in CRP from baseline remained greater in the intervention group (*P *= 0.034; Supplementary Tables 5 and 6).

When participants were stratified by baseline glycemic control, a significantly greater reduction in HbA1c at 3 months was observed in the poorly controlled subgroup (HbA1c ≥ 6.5%) compared with the well-controlled subgroup (HbA1c < 6.5%) within the intervention group (*P* = 0.004). No significant subgroup differences were detected at 6 months or in the control group at either time point (Supplementary Tables 7 and 8).

### Effects of nonsurgical periodontal therapy on the oral microbiome

2,776,170 sequences were produced in total, averaging 11,017 per sample (range: 6,741 to 18,545) after quality filtering. The majority of the sequences had a length ranging from 1434 to 1485 bp. The rarefaction analysis was used to estimate the species richness of the microbiota in saliva and GCF samples of each participant (Figure S1).

#### *α*-diversity and *β*-diversity analysis of the oral microbiome before and after nonsurgical periodontal therapy

**Figure 2.**
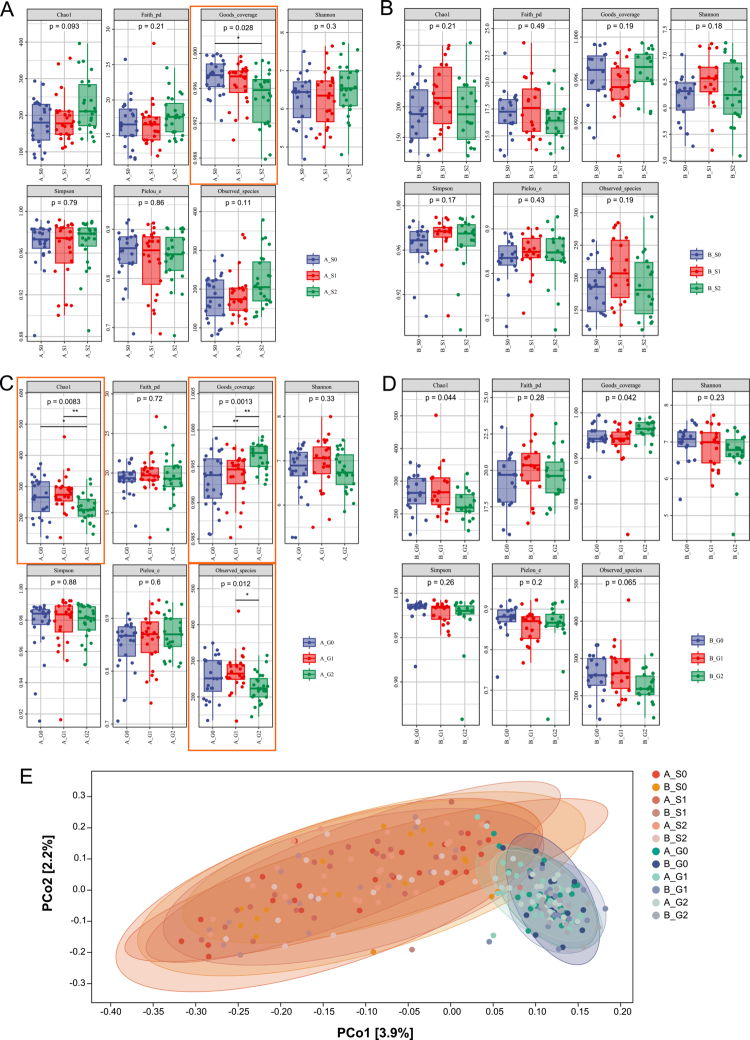
Alpha diversity and beta diversity analysis. **(A-B)** *α*-diversity analysis of the salivary microbiome. *p < 0.05, **p < 0.01. **(C-D)** *α*-diversity analysis of the gingival crevicular fluid (GCF) microbiome. *p < 0.05, **p < 0.01. **(E)** Principal Coordinate Analysis (PCoA) based on Bray-Curtis distance showing the variation in community structure of salivary and GCF microbiota across the 12 groups. S, saliva; G, GCF; group A, intervention group; group B, control group; T0, baseline; T1, 3-month follow-up; T2, 6-month follow-up.

We used seven *α*-diversity metrics to characterise within-sample community structure (Figure 2). Chao1 and Observed_species quantify richness (Observed_species is the number of observed features/ASVs, whereas Chao1 estimates total richness by accounting for rare features, particularly singletons and doubletons). Shannon and Simpson quantify diversity by jointly considering richness and relative abundance (Shannon is entropy-based and sensitive to both richness and evenness; Simpson is dominance-weighted and gives more weight to abundant taxa). Pielou_e quantifies evenness (Shannon normalised by ln(richness), ranging from 0 to 1). Faith_pd quantifies phylogenetic diversity as the total branch length spanned by observed taxa in a sample. Goods_coverage (Good’s coverage estimator) reflects sampling completeness as a normalised index ranging from 0 to 1, commonly computed as 1 − (F1/N), where F1 is the number of singleton features and *N* is the total number of reads/sequences.

*α*-diversity of the salivary microbial community at baseline and at the 3- and 6-month follow-up visits is shown in Figure 2. In the intervention group, Goods_coverage showed a gradual decline over time, with a statistically significant difference between baseline and 6 months (Figure 2A). Goods_coverage values closer to 1 indicate higher sampling completeness (i.e. a lower proportion of singletons), and decreases may indicate an increased proportion of rare/singleton features rather than ‘absolute’ coverage of all taxa present. No *α*-diversity indices differed significantly over time in the control group (Figure 2B). *α*-diversity analysis of the GCF microbiota is shown in Figure 2. In the intervention group, Chao1 and Goods_coverage differed significantly between baseline and both follow-up time points, while Observed_species differed between 3 and 6 months (Figure 2C). No significant longitudinal changes in *α*-diversity were observed in the control group (Figure 2D).

Principal Coordinate Analysis (PCoA) was performed using Bray-Curtis distances to assess *β*-diversity (Figure 2E). It showed no marked temporal shifts in microbial community structure in saliva or GCF in either group across follow-up time points. In contrast, clear separation was observed between saliva- and GCF-derived microbial communities, indicating distinct microbial profiles across these oral niches.

#### Longitudinal changes in the oral microbiome following nonsurgical periodontal therapy

**Figure 3.**
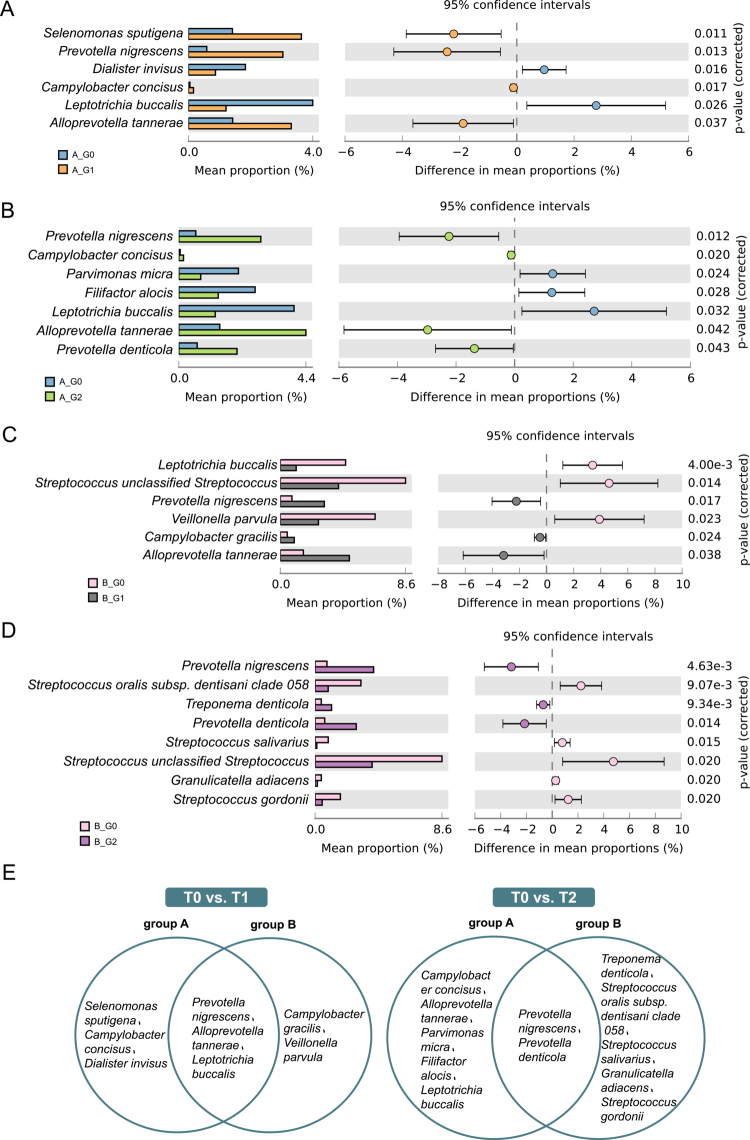
Comparison of the relative abundance of bacterial species in gingival crevicular fluid (GCF) between time points. **(A)** Group A: 3 months after non-surgical periodontal therapy (NSPT) versus baseline. **(B)** Group A: 6 months after NSPT versus baseline. **(C)** Group B: 3-month follow-up versus baseline. **(D)** Group B: 6-month follow-up versus baseline. **(E)** Venn diagram showing differential microbial taxa in GCF between the intervention group and the control group. Group A, intervention group; group B, control group; T0, baseline; T1, 3-month follow-up; T2, 6-month follow-up. *P *< 0.05 by Welch’s t-test.

For both saliva and GCF, taxa with a median relative abundance  >  0.1% were included in differential analysis using Welch’s t test. In GCF, multiple taxa showed longitudinal changes after nonsurgical periodontal therapy in both groups (Figure 3). In the intervention group, distinct sets of taxa differed between baseline and 3 months and between baseline and 6 months, with some taxa showing reduced abundance over time and others showing increased abundance. Similar longitudinal patterns were observed in the control group; however, the taxa involved and their temporal trajectories differed from those observed in the intervention group, as illustrated by the Venn diagram (Figure 3E).

**Figure 4.**
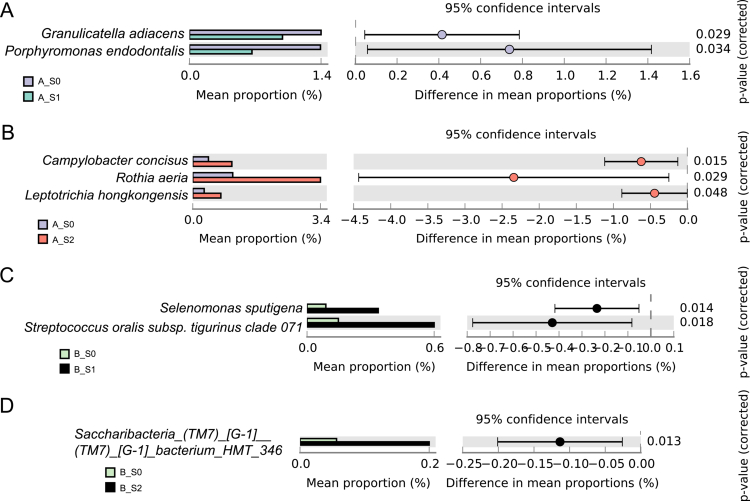
Comparison of the relative abundance of bacterial species in saliva between time points. **(A)** Group A: 3 months after non-surgical periodontal therapy (NSPT) versus baseline. **(B)** Group A: 6 months after NSPT versus baseline. **(C)** Group B: 3-month follow-up versus baseline. **(D)** Group B: 6-month follow-up versus baseline. Group A, intervention group; group B, control group; T0, baseline; T1, 3-month follow-up; T2, 6-month follow-up. *P *< 0.05 by Welch’s t-test.

In saliva, fewer taxa exhibited longitudinal variation compared with GCF (Figure 4). In the intervention group, only a limited number of taxa differed between baseline and follow-up at 3 and 6 months, whereas different taxa showed temporal changes in the control group. Overall, the salivary microbiome displayed more limited temporal variation than the GCF microbiome following nonsurgical periodontal therapy.

After FDR correction for multiple testing, no individual taxa remained statistically significant.

### Longitudinal changes in the oral metabolome after nonsurgical periodontal therapy

**Figure 5.**
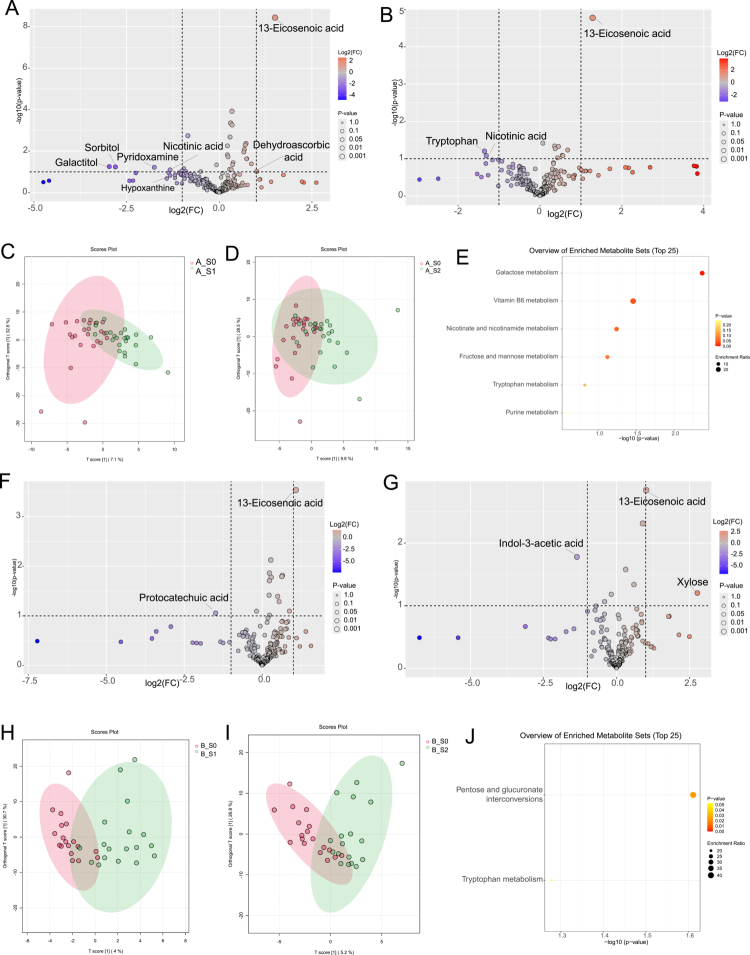
Comprehensive analysis of saliva metabolites. **(A, B, F, G)** Volcano plot showing differential metabolites between groups, based on *p *< 0.1 and |log2(FC)| > 1. **(A)** Group A: 3 months after non-surgical periodontal therapy (NSPT) versus baseline. **(B)** Group A: 6 months after NSPT versus baseline. **(F)** Group B: 3-month follow-up versus baseline. **(G)** Group B: 6-month follow-up versus baseline. **(C, D, H, I)** PLS-DA of the metabolic profiles of saliva samples. PLS-DA, partial least squares discriminant analysis. **(E, J)** KEGG enrichment analysis of the differential metabolites in group A and B, respectively. Group A, intervention group; group B, control group; T0, baseline; T1, 3-month follow-up; T2, 6-month follow-up.

After preprocessing of the salivary GC-MS widely targeted metabolomics dataset (removal of putative false-positive peaks and internal standards, and peak merging), 257 metabolites were retained for downstream analyses. Changes before and after nonsurgical periodontal therapy were initially screened using fold-change analysis together with a two-sided Student’s t test (|log2FC|  >  1 and nominal *P *< 0.1; Figure 5A, B, F, G). Partial least squares discriminant analysis (PLS-DA) of salivary metabolites in the intervention group showed a more pronounced separation at 3 months (Figure 5C) than at 6 months (Figure 5D). PLS-DA of salivary metabolites in the control group indicated a more evident separation at 6 months (Figure 5I) than at 3 months (Figure 5H).

To account for multiple testing, *P* values from the univariate analyses were adjusted using the Benjamini-Hochberg false discovery rate (BH-FDR) procedure. After FDR correction (q < 0.25), only a very limited signal remained: 13-eicosenoic acid consistently showed increased levels at both 3 and 6 months relative to baseline in both the intervention and control groups (Supplementary Table S9).

Pathway enrichment based on nominally altered metabolites suggested that the intervention group showed broader pathway coverage, including galactose metabolism, vitamin B6 metabolism, nicotinate and nicotinamide metabolism, fructose and mannose metabolism, tryptophan metabolism, and purine metabolism (Figure 5E). Whereas the control group exhibited enrichment in fewer pathways: pentose and glucuronate interconversions and tryptophan metabolism (Figure 5J).

### Correlation analysis between omics changes and clinical changes

**Figure 6.**
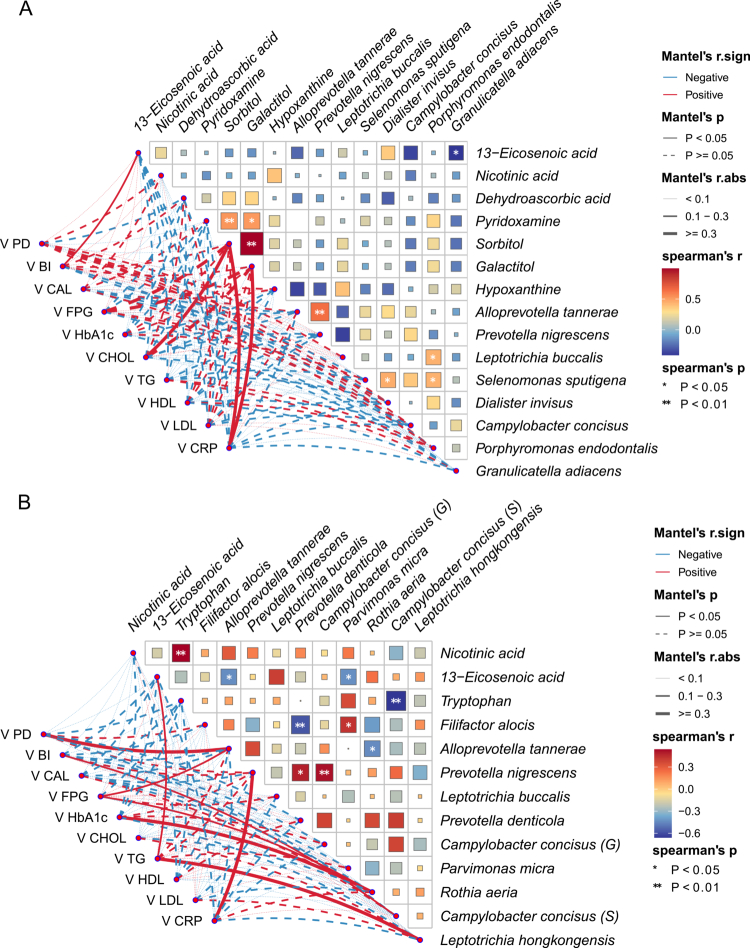
Correlation heatmap and network analysis linking changes in differential metabolites and differential microbes with changes in clinical parameters. **(A)** Three months after non-surgical periodontal therapy in the treatment group. **(B)** Six months after non-surgical periodontal therapy in the treatment group. Solid lines indicate *P *< 0.05, and dashed lines indicate *P* ≥ 0.05. Red denotes positive correlations and blue denotes negative correlations. *Significant correlation between the species and metabolites (*P < 0.05, **P < 0.01, ***P < 0.001).

**Figure 7.**
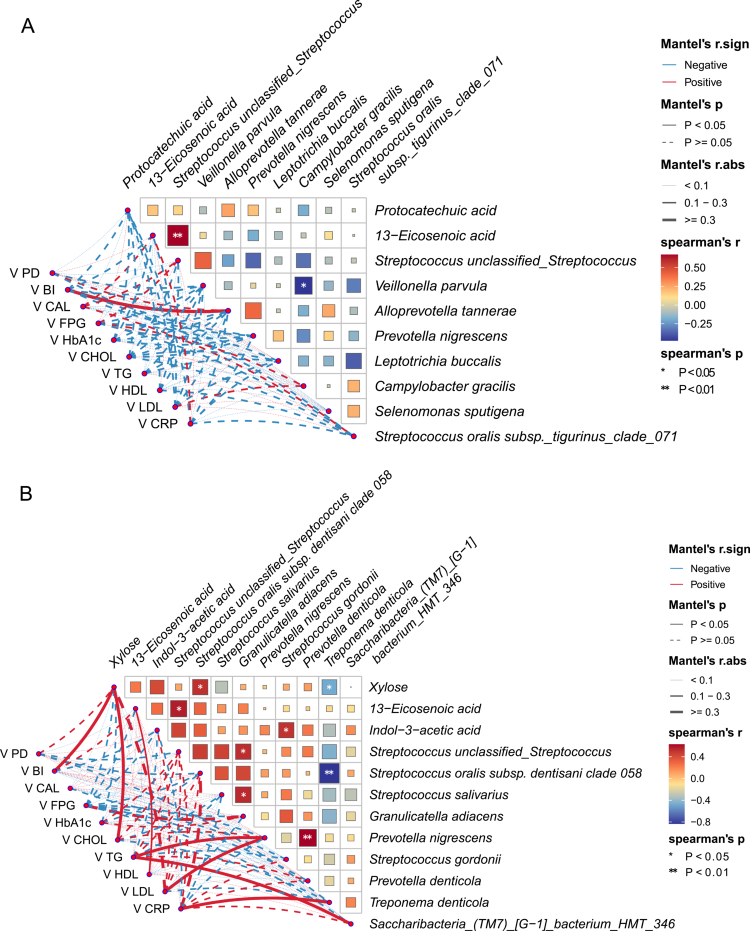
Correlation heatmap and network analysis showing associations between changes in differential metabolites and differential microbes and changes in clinical parameters. **(A)** Three-month follow-up in the control group. **(B)** Six-month follow-up in the control group. Solid lines indicate *P *< 0.05, and dashed lines indicate *P*≥0.05. Red denotes positive correlations and blue denotes negative correlations. *Significant correlation between the species and metabolites (*P < 0.05, **P < 0.01, ***P < 0.001).

Associations between changes (Δ) in selected taxa/metabolites and changes in clinical indices were explored using Mantel tests (Figures 6-7). In the intervention group at 3 months, Δsorbitol and Δgalactitol showed positive associations with ΔCRP, and were positively correlated with each other, suggesting coordinated metabolite shifts linked to systemic inflammatory change (Figure 6A). At 6 months, cross-domain co-variation patterns were observed between changes in specific taxa/metabolites and periodontal or glycemic indices (Figure 6B).

In the control group, limited cross-domain associations were observed at 3 months, whereas broader co-variation patterns were evident at 6 months, including associations involving inflammatory and lipid-related indices (Figure 7A, B). Overall correlation patterns are visualised in Figures 6–7.

### KEGG pathway annotation of prioritised taxa and metabolites

Gene information for prioritised taxa was retrieved from NCBI. Based on the Mantel correlation results, representative taxa and metabolites were selected for functional contextualisation. KEGG pathway mapping was performed using KEGG Mapper, yielding annotations spanning over 200 pathways. Pathways of interest were then summarised with reference to prior literature.

Glutathione metabolism emerged as a recurrent context in the 3-month intervention group. Dehydroascorbic acid (DHA) and genes from multiple prioritised taxa could be mapped to this pathway (Figure S2). As DHA participates in the glutathione redox cycle, this mapping provides a functional framework for interpreting the observed microbe-metabolite patterns in relation to redox homoeostasis (Figure S3).

Purine metabolism was also highlighted at 3 months. Hypoxanthine and genes from the same set of prioritised taxa were mapped to the purine metabolism pathway (Figure S4), offering a pathway-level context for the observed associations. Given the role of hypoxanthine in purine catabolism and its link to oxidative processes, this pathway may be relevant to the inflammatory milieu (Figure S5).

At 6 months in the intervention group, tryptophan and *Campylobacter concisus* were mapped to multiple KEGG pathways, including phenylalanine, tyrosine and tryptophan biosynthesis and biosynthesis of cofactors (Figure S6).

## Discussion

In patients with T2DM and periodontitis, dysregulated microbe-metabolite-host interactions may exacerbate periodontal tissue destruction and adversely affect systemic metabolism. However, the impact of NSPT on the oral microbial community and its associated metabolite profiles remains incompletely understood. Therefore, this study integrates microbiome and metabolome analyses to systematically characterise microbiome-metabolome features in T2DM patients with periodontitis before and after NSPT, to investigate their associations with periodontal clinical parameters and systemic indicators, and to identify potential intervention targets for T2DM-associated periodontitis.

In the present study, baseline BI, HDL, and CRP levels were significantly higher in the intervention group than in the control group (*P* = 0.041, *P* = 0.039, and *P* = 0.032, respectively), suggesting a greater periodontal and systemic inflammatory burden in the intervention group, possibly attributable to differential loss to follow-up during the study. To mitigate potential bias related to baseline disease severity or regression to the mean, we performed ANCOVA analyses adjusting for baseline values (Table S2). Importantly, the intervention effect on periodontal inflammation, as reflected by BI, remained significant after adjustment, supporting a true treatment-related effect rather than a statistical artefact. A similar pattern was observed for CRP at the 3-month follow-up, whereas metabolic parameter HDL did not retain significant group differences after adjustment. Despite ANCOVA adjustment, baseline imbalance between groups may have introduced residual confounding. In particular, the higher baseline BI, HDL, and CRP levels in the intervention group raise the possibility that part of the observed improvements may reflect regression to the mean rather than a pure treatment effect. Moreover, improvements in PD and BI were greater in the intervention group than in the control group (Table S3, S5). These findings suggest that the intervention primarily exerted its effects on local periodontal inflammation and short-term systemic inflammatory burden.

For glyco-lipid metabolic parameters and systemic inflammatory markers, both groups showed varying degrees of improvement in selected indices at 3 and 6 months. However, no significant change in HbA1c was observed. After stratifying the intervention and control groups according to baseline glycemic control, participants in the intervention group with poor glycemic control (HbA1c ≥ 6.5%) exhibited a significantly greater reduction in HbA1c at 3 months than those with good glycemic control (HbA1c < 6.5%) (*P* = 0.004). No such difference was observed at 6 months in the intervention group or at either time point in the control group. These findings suggest that intensive NSPT may yield a more pronounced improvement in HbA1c among patients with suboptimal glycemic control at baseline, with the effect being more evident at 3 months, broadly consistent with previous studies [10–14]. Nevertheless, because the stratified subgroups were small, the clinical significance of this observation warrants confirmation in larger cohorts. From a clinical perspective, the observed systemic metabolic improvements were modest and largely non-significant, indicating that the primary benefit of NSPT in this cohort was related to periodontal inflammation rather than systemic metabolic control. Therefore, NSPT should be viewed primarily as a periodontal therapy that improves oral health, with potential but inconsistent adjunctive metabolic benefits.

Considering the use of stringent sequence data processing and quality control, we interpret the results of *α*-diversity as reflecting biologically meaningful changes in the microbial community. *α*-diversity analysis of the oral microbiome suggested that NSPT was accompanied by changes in within-sample community characteristics in the intervention group. In saliva, the Goods_coverage index differed at the 6-month follow-up compared with baseline. In GCF, several indices reflecting richness and observed community membership (Chao1, Goods_coverage, and Observed_species) also differed after therapy relative to baseline. In contrast, none of the *α*-diversity metrics showed comparable temporal changes in the control group. Together, these findings imply that NSPT may modulate the oral microbial ecosystem in patients with T2DM, with the signal being more evident in GCF than in saliva. This is biologically plausible given that GCF is more proximal to the periodontal pocket environment and may therefore respond more readily to changes in subgingival biofilm burden and local inflammation following treatment.

Consistent with the *β*-diversity results, the overall community structure appeared relatively stable over time within each niche, whereas saliva and GCF remained clearly segregated, highlighting strong site-specificity of the oral microbiome and suggesting that niche differences may outweigh short-term temporal changes following therapy.

For the microbiome, univariate screening suggested time-associated shifts in the relative abundance of several taxa in GCF following comprehensive NSPT. Similar temporal changes were also observed in the control group, which may reflect background variability of the subgingival ecosystem over time. Notably, the sets of taxa showing nominal changes were largely non-overlapping between the intervention and control groups at both follow-up time points, indicating group-specific temporal profiles in GCF. In saliva, only one to two taxa showed nominal changes from baseline in each group, consistent with the generally greater stability of salivary microbiota compared with GCF.

BI reflects local periodontal inflammation. Changes in 13-eicosenoic acid, xylose, *Rothia aeria*, and *Alloprevotella tannerae* were positively correlated with changes in BI, suggesting that these metabolites and/or taxa may be involved in a bidirectional interaction with periodontal inflammatory status. CRP is a marker of systemic inflammation; metabolites and taxa showing positive correlations with changes in CRP included sorbitol, galactitol, *Prevotella nigrescens*, and *Treponema denticola*, implying potential associations with an enhanced systemic inflammatory response. Other metabolites or taxa that were correlated with glyco-lipid metabolic indices may represent candidate biomarkers related to glucose and lipid metabolism.

At both the 3- and 6-month follow-up visits, salivary levels of 13-eicosenoic acid increased significantly in both the intervention and control groups. Previous studies have suggested that eicosenoic acid, an unsaturated fatty acid that is relatively abundant in natural oils, may reduce the risk of T2DM [27] and protect insulin-producing cells in rats from the lipotoxic effects of saturated fatty acids [28]. Therefore, an increase in 13-eicosenoic acid may be beneficial for improving insulin resistance in patients with T2DM. In the present study, in the control group at 6 months, the change in 13-eicosenoic acid was significantly positively correlated with the change in HDL, suggesting a potential favourable association with glyco-lipid metabolism.

Three months after NSPT, salivary DHA levels increased in the intervention group. A previous study reported elevated DHA levels in the GCF of patients with generalised aggressive periodontitis compared with healthy individuals [29]. In patients with diabetes, reduced erythrocyte ascorbic acid concentrations may increase erythrocyte fragility and ultimately lead to localised haemolysis. Because ascorbic acid can be regenerated following uptake of DHA into erythrocytes and subsequent reduction, increased DHA levels may help alleviate diabetes-associated haemolysis [30]. In our KEGG pathway annotation analysis, DHA and genes from multiple taxa were co-mapped to the glutathione metabolism pathway. The relationship between DHA and glutathione (GSH) metabolism primarily involves redox reactions: DHA interacts with GSH to regenerate ascorbic acid and restore its antioxidant capacity while producing oxidised glutathione (GSSG). Glutathione reductase then reduces GSSG back to GSH, thereby restoring GSH activity. This interplay is critical for maintaining intracellular antioxidant homoeostasis and enhancing cellular resistance to oxidative stress, potentially reducing the risk of multiple diseases associated with oxidative damage [31]. Of course, we need to emphasise that these pathway interpretations are derived from correlation analyses and KEGG annotations; they do not demonstrate causality. In the absence of functional validation or targeted biochemical measurements, these findings should be interpreted as exploratory rather than as definitive mechanistic evidence.

Three months after NSPT, salivary hypoxanthine levels decreased in the intervention group. KEGG pathway annotation mapped hypoxanthine together with multiple taxa to the purine metabolism pathway. Hypoxanthine participates in purine metabolism and is ultimately converted to uric acid with concomitant generation of ROS [32]. Free radicals produced during this process (e.g. superoxide) can exacerbate oxidative stress. Oxidative stress plays an important role in the pathogenesis of T2DM and can worsen insulin resistance. In periodontal inflammation, enhanced local oxidative stress promotes the conversion of hypoxanthine to uric acid via purine metabolism; uric acid, in turn, can aggravate inflammation and further increase free radical production, thereby accelerating hypoxanthine metabolism and exacerbating periodontal tissue damage, creating a vicious cycle. As with the glutathione pathway, the proposed link between purine metabolism and oxidative stress in this context remains speculative and requires experimental verification.

At 6 months after NSPT, the metabolite tryptophan and the taxon *Campylobacter concisus* could be co-mapped to multiple KEGG pathways. Their changes were negatively correlated, and salivary tryptophan levels were decreased. Tryptophan is an essential amino acid that is critical for protein biosynthesis and serves as a precursor for a range of bioactive compounds. It influences multiple pathophysiological processes, including neuronal function, metabolism, inflammatory responses, oxidative stress, immune regulation, and gut homoeostasis, and has been extensively studied in various diseases, such as gastrointestinal and neurological disorders [33]. However, its role in diabetes and periodontitis has been far less explored. One study reported tryptophan as a differential metabolite in the metabolomic profiles of subgingival plaque when comparing individuals with periodontitis versus healthy controls, as well as patients with T2DM with or without periodontitis [34]. Emerging evidence supports a close link between dysregulated tryptophan metabolism and disease: levels or ratios of tryptophan metabolites are significantly associated with multiple clinical features, and modulation of tryptophan metabolism may help control disease progression [35,36]. Moreover, tryptophan metabolites detected via the kynurenine pathway in plasma and saliva have been proposed as sensitive biomarkers of oxidative stress status [37].

At the 3-month follow-up in the control group, salivary protocatechuic acid levels decreased. Protocatechuic acid has been shown to suppress lipopolysaccharide (LPS)-induced production of IL-6 and IL-8 in human gingival fibroblasts and to inhibit LPS-triggered NF-κB activation. Its anti-inflammatory effects have been linked to activation of the peroxisome proliferator–activated receptor-*γ* (PPAR-*γ*) pathway, thereby attenuating LPS-induced inflammatory responses [38]. In addition, protocatechuic acid has been reported to ameliorate high-fat diet-induced obesity and insulin resistance in mice [39]. At the 6-month follow-up in the control group, salivary indole-3-acetic acid levels also decreased. Indole-3-acetic acid has been identified as a key bioactive metabolite capable of improving insulin resistance by activating the intestinal aryl hydrocarbon receptor, enhancing gut barrier function, and reducing systemic inflammation, thereby alleviating insulin resistance [40].

In summary, following NSPT, the intervention group showed increased salivary levels of metabolites potentially conducive to glycemic control and improved insulin sensitivity, along with decreased levels of metabolites that may exacerbate oxidative stress and inflammatory responses. These changes may help explain the improvement in glycemic control observed in patients with T2DM and periodontitis after therapy. By contrast, the reduction of metabolites associated with improved insulin sensitivity in the control group may be related to suboptimal glycemic control in patients with longstanding periodontal inflammation. Nevertheless, the associations and putative mechanisms described above require further validation through in vitro and experimental studies.

However, the above conclusions should be interpreted in light of the study limitations. First, to minimise the influence of confounding factors, we applied stringent inclusion and exclusion criteria, which resulted in relatively small sample sizes in our study. Accordingly, our findings represent a preliminary exploration of oral microbiome and metabolome features in the population meeting these criteria. Importantly, after FDR correction, no individual microbial taxon remained statistically significant. This underscores the exploratory nature of our microbiome analyses and indicates that the nominal associations reported should not be overinterpreted as definitive treatment-induced shifts. Given the high dimensionality of the omics data and the modest sample size, feature-level findings should be interpreted cautiously and primarily as hypothesis-generating, and further validation in larger, multicenter cohorts is warranted. Second, because the volume of GCF obtainable was limited and insufficient for simultaneous microbiome and metabolome profiling, only the GCF microbiome was analysed, and metabolomic characterisation was not performed. Given that GCF is the niche most closely related to periodontitis, the lack of GCF metabolomic data represents a major gap in the integrative analyses; future studies should endeavour to ensure complete profiling of both the microbiome and metabolome in GCF, possibly by using more sensitive analytical platforms (e.g. nanoflow LC-MS) or pooling samples from multiple sites to obtain sufficient volume for metabolomic profiling. Additionally, the integration of microbiome and metabolome data in this study relies on correlation-based approaches, which provide only preliminary insights into potential interactions and do not support causal inference. More advanced integrative frameworks, such as sparse canonical correlation analysis or Bayesian network modelling, should be required in future studies with larger sample sizes. Finally, the microbe-metabolite association analyses in this study relied primarily on KEGG-based annotations, providing indirect evidence for functional mechanisms. We did not incorporate metagenomic or metatranscriptomic approaches, and several pathway-related hypotheses proposed here lack in vitro functional validation. Future work should build upon our findings to strengthen and extend the mechanistic evidence.

## Conclusion

In this longitudinal multi-omics study of patients with T2DM and periodontitis, comprehensive NSPT significantly improved periodontal inflammatory status and modestly influenced systemic inflammatory and glycemic markers, with a more pronounced HbA1c reduction observed in those with poor baseline glycemic control. Integrated microbiome and metabolome analyses revealed that NSPT was associated with shifts in oral microbial diversity and metabolite profiles, with enrichment in redox-related pathways, particularly glutathione and purine metabolism. Together, these findings extend current understanding of the relationship between periodontitis and T2DM by suggesting that oral microbe-metabolite alterations may accompany, and potentially be associated with, the interplay between local periodontal inflammation and systemic metabolic dysregulation. These exploratory findings suggest that oxidative stress pathways may represent a biological link between periodontal therapy and systemic metabolic changes in T2DM. From a translational perspective, several candidate metabolites (e.g. 13-eicosenoic acid, sorbitol, tryptophan) and taxa (e.g. *Rothia aeria*, *Treponema denticola*, *Campylobacter concisus*) identified here warrant further validation in larger cohorts and mechanistic studies. Given the exploratory nature and sample size limitations, our results should be interpreted as hypothesis-generating rather than confirmatory. Future research should prioritise functional validation of the implicated pathways and adequately powered trials to determine whether NSPT confers clinically meaningful systemic benefits in T2DM patients.

## Supplementary Material

Supplementary material.docx Supplementary material.docx

## Data Availability

The raw sequencing data from our study can be accessed in the NCBI Sequence Read Archive under the accession number PRJNA1088636 and PRJNA1372956.

## References

[1] Petersen PE, Ogawa H. The global burden of periodontal disease: towards integration with chronic disease prevention and control. Periodontol 2000. 2012;60(1):15–39. doi: 10.1111/j.1600-0757.2011.00425.x 22909104

[2] Graziani F, Gennai S, Solini A, et al. A systematic review and meta-analysis of epidemiologic observational evidence on the effect of periodontitis on diabetes an update of the EFP-AAP review. J Clin Periodontol. 2018;45(2):167–187. doi: 10.1111/jcpe.12837 29277926

[3] Borgnakke WS, Ylöstalo PV, Taylor GW, et al. Effect of periodontal disease on diabetes: systematic review of epidemiologic observational evidence. J Clin Periodontol. 2013;40(Suppl 14):S135–S152. doi: 10.1111/jcpe.12080 23627324

[4] Graves DT, Levine M, Aldosary S, et al. Understanding the periodontitis-diabetes linkage: mechanisms and evidence. J Dent Res. 2026;105(1):21–30. doi: 10.1177/00220345251388340 41292096 PMC12701909

[5] Montero E, Bujaldón R, Montanya E, et al. Cross-sectional association between severe periodontitis and diabetes mellitus: a nation-wide cohort study. J Clin Periodontol. 2024;51(4):368–379. doi: 10.1111/jcpe.13937 38140803

[6] Morita I, Inagaki K, Nakamura F, et al. Relationship between periodontal status and levels of glycated hemoglobin. J Dent Res. 2012;91(2):161–166. doi: 10.1177/0022034511431583 22157098

[7] Polak D, Shapira L. An update on the evidence for pathogenic mechanisms that May link periodontitis and diabetes. J Clin Periodontol. 2018;45(2):150–166. doi: 10.1111/jcpe.12803 29280184

[8] Madianos PN, Koromantzos PA. An update of the evidence on the potential impact of periodontal therapy on diabetes outcomes. J Clin Periodontol. 2018;45(2):188–195. doi: 10.1111/jcpe.12836 29277978

[9] Engebretson SP, Hyman LG, Michalowicz BS, et al. The effect of nonsurgical periodontal therapy on hemoglobin A1c levels in persons with type 2 diabetes and chronic periodontitis: a randomized clinical trial. J Am Med Assoc. 2013;310(23):2523–2532. doi: 10.1001/jama.2013.282431 PMC4089989 24346989

[10] Simpson TC, Weldon JC, Worthington HV, et al. Treatment of periodontal disease for glycaemic control in people with diabetes mellitus. Cochrane Database Syst Rev. 2015;2015(11):Cd004714. doi: 10.1002/14651858.CD004714.pub3 26545069 PMC6486035

[11] Simpson TC, Needleman I, Wild SH, et al. Treatment of periodontal disease for glycaemic control in people with diabetes. Cochrane Database Syst Rev. 2010;(5):Cd004714. doi: 10.1002/14651858.CD004714.pub2 20464734

[12] Spangler L, Reid RJ, Inge R, et al. Cross-sectional study of periodontal care and glycosylated hemoglobin in an insured population. Diabetes Care. 2010;33(8):1753–1758. doi: 10.2337/dc09-1412 20504894 PMC2909057

[13] Simpson TC, Clarkson JE, Worthington HV, et al. Treatment of periodontitis for glycaemic control in people with diabetes mellitus. Cochrane Database Syst Rev. 2022;4(4):Cd004714. doi: 10.1002/14651858.CD004714.pub4 35420698 PMC9009294

[14] Chee HK, Tan S, Tjakkes G, et al. Long-term effect of periodontal therapy on HbA1c changes in type 2 diabetes. J Dent Res. 2026;105(1):67–76. doi: 10.1177/00220345251357875 40817763

[15] Artese HP, Foz AM, Rabelo M, et al. Periodontal therapy and systemic inflammation in type 2 diabetes mellitus: a meta-analysis. PLoS One. 2015;10(5):e0128344. doi: 10.1371/journal.pone.0128344 26010492 PMC4444100

[16] Genco RJ, Graziani F, Hasturk H. Effects of periodontal disease on glycemic control, complications, and incidence of diabetes mellitus. Periodontol 2000. 2020;83(1):59–65. doi: 10.1111/prd.12271 32385875

[17] Shi B, Lux R, Klokkevold P, et al. The subgingival microbiome associated with periodontitis in type 2 diabetes mellitus. ISME J. 2020;14(2):519–530. doi: 10.1038/s41396-019-0544-3 31673077 PMC6976570

[18] Silva-Boghossian CM, Orrico SRP, Gonçalves D, et al. Microbiological changes after periodontal therapy in diabetic patients with inadequate metabolic control. Braz Oral Res. 2014;28:1–9. doi: 10.1590/1807-3107BOR-2014.vol28.0007 24918369

[19] Bauermeister A, Mannochio-Russo H, Costa-Lotufo LV, et al. Mass spectrometry-based metabolomics in microbiome investigations. Nat Rev Microbiol. 2022;20(3):143–160. doi: 10.1038/s41579-021-00621-9 34552265 PMC9578303

[20] Califf KJ, Schwarzberg-Lipson K, Garg N, et al. Multi-omics analysis of periodontal pocket microbial communities Pre- and posttreatment. mSystems. 2017;2(3). doi: 10.1128/mSystems.00016-17 PMC5513737 28744486

[21] Liebsch C, Pitchika V, Pink C, et al. The saliva metabolome in association to oral health status. J Dent Res. 2019;98(6):642–651. doi: 10.1177/0022034519842853 31026179

[22] Barnes VM, Ciancio S, Shibly O, et al. Metabolomics reveals elevated macromolecular degradation in periodontal disease. J Dent Res. 2011;90(11):1293–1297. doi: 10.1177/0022034511416240 21856966

[23] Romano F, Meoni G, Manavella V, et al. Effect of non-surgical periodontal therapy on salivary metabolic fingerprint of generalized chronic periodontitis using nuclear magnetic resonance spectroscopy. Arch Oral Biol. 2019;97:208–214. doi: 10.1016/j.archoralbio.2018.10.023 30396039

[24] Takahashi N. Oral microbiome metabolism: from ‘Who are They?’ to "What are they doing? J Dent Res. 2015;94(12):1628–1637. doi: 10.1177/0022034515606045 26377570

[25] Baima G, Iaderosa G, Citterio F, et al. Salivary metabolomics for the diagnosis of periodontal diseases: a systematic review with methodological quality assessment. Metabolomics. 2021;17(1):1. doi: 10.1007/s11306-020-01754-3 33387070

[26] Kuboniwa M, Sakanaka A, Hashino E, et al. Prediction of periodontal inflammation via metabolic profiling of saliva. J Dent Res. 2016;95(12):1381–1386. doi: 10.1177/0022034516661142 27470067

[27] Zhu X, Chen L, Lin J, et al. Association between fatty acids and the risk of impaired glucose tolerance and type 2 diabetes mellitus in American adults: NHANES 2005-2016. Nutr Diabetes. 2023;13(1):8. doi: 10.1038/s41387-023-00236-4 37127641 PMC10151340

[28] Plötz T, Hartmann M, Lenzen S, et al. The role of lipid droplet formation in the protection of unsaturated fatty acids against palmitic acid induced lipotoxicity to rat insulin-producing cells. Nutr Metab (Lond). 2016;13:16. doi: 10.1186/s12986-016-0076-z 26918025 PMC4766664

[29] Chen HW, Zhou W, Liao Y, et al. Analysis of metabolic profiles of generalized aggressive periodontitis. J Periodontal Res. 2018;53(5):894–901. doi: 10.1111/jre.12579 29974463

[30] Tu H, Li H, Wang Y, et al. Low red blood cell vitamin C concentrations induce red blood cell fragility: a link to diabetes via glucose, glucose transporters, and dehydroascorbic acid. EBioMedicine. 2015;2(11):1735–1750. doi: 10.1016/j.ebiom.2015.09.049 26870799 PMC4740302

[31] Foyer CH, Kunert K. The ascorbate-glutathione cycle coming of age. J Exp Bot. 2024;75(9):2682–2699. doi: 10.1093/jxb/erae023 38243395 PMC11066808

[32] Wen S, Arakawa H, Tamai I. Uric acid in health and disease: from physiological functions to pathogenic mechanisms. Pharmacol Ther. 2024;256:108615. doi: 10.1016/j.pharmthera.2024.108615 38382882

[33] Xue C, Li G, Zheng Q, et al. Tryptophan metabolism in health and disease. Cell Metab. 2023;35(8):1304–1326. doi: 10.1016/j.cmet.2023.06.004 37352864

[34] Jiang L, Zhang J, Fang M, et al. Analysis of subgingival micro-organisms based on multi-omics and Treg/Th17 balance in type 2 diabetes with/without periodontitis. Front Microbiol. 2022;13:939608. doi: 10.3389/fmicb.2022.939608 36519166 PMC9743466

[35] Cheng S, Song S, Zhang W, et al. Tryptophan-derived microbial metabolite Indole-3-Acetic acid ameliorates periodontitis through AhR/CYP1A1-Mediated macrophage polarisation. J Clin Periodontol. 2025. 10.1111/jcpe.70064 PMC12972604 41285120

[36] Ding J, Tan L, Wu L, et al. Regulation of tryptophan-indole metabolic pathway in porphyromonas gingivalis virulence and microbiota dysbiosis in periodontitis. NPJ Biofilms Microbiomes. 2025;11(1):37. doi: 10.1038/s41522-025-00669-y 40011497 PMC11865485

[37] Buczko P, Zalewska A, Szarmach I. Saliva and oxidative stress in oral cavity and in some systemic disorders. J Physiol Pharmacol. 2015;66(1):3–9. 25716960

[38] Wang Y, Zhou J, Fu S. Preventive effects of protocatechuic acid on LPS-Induced inflammatory response in human gingival fibroblasts via activating PPAR-γ. Inflammation. 2015;38(3):1080–1084. doi: 10.1007/s10753-014-0073-1 25433806

[39] Xiang Y, Huang R, Wang Y, et al. Protocatechuic acid ameliorates high fat diet-induced obesity and insulin resistance in mice. Mol Nutr Food Res. 2023;67(3):e2200244. doi: 10.1002/mnfr.202200244 36285395

[40] Nie Q, Sun Y, Hu W, et al. Glucomannan promotes bacteroides ovatus to improve intestinal barrier function and ameliorate insulin resistance. Imeta. 2024;3(1):e163. doi: 10.1002/imt2.163 38868507 PMC10989147

