# Supplementary material for: Nonsurgical periodontal therapy remodels oral microbiome-metabolome networks and associates with glycemic and inflammatory improvements in type 2 diabetes mellitus with periodontitis: a 6-month longitudinal study
Source: J Oral Microbiol. 2026 Apr 17;18(1):2660482. doi: 10.1080/20002297.2026.2660482 (PMC13094239; doi:10.1080/20002297.2026.2660482)
Supplement: Supplementary material.docx [file ZJOM_A_2660482_SM8337.docx]

**Nonsurgical periodontal therapy remodels oral microbiome-metabolome networks and associates with glycemic and inflammatory improvements in type 2 diabetes mellitus with periodontitis: A 6-month longitudinal study**

**Jing Diao^†, 1^, Hejia Li^†, 2^, Shuguo Zheng^*, 1^, Jiahui Niu^*, 3^, Chao Yuan^*, 1^**

^1^Department of Preventive Dentistry, Peking University School and Hospital of Stomatology & National Center for Stomatology & National Clinical Research Center for Oral Diseases & National Engineering Research Center of Oral Biomaterials and Digital Medical Devices& Beijing Key Laboratory of Digital Stomatology, Beijing, PR China

^2^Third Clinical Division, Peking University School and Hospital of Stomatology & National Center for Stomatology & National Clinical Research Center for Oral Diseases & National Engineering Research Center of Oral Biomaterials and Digital Medical Devices& Beijing Key Laboratory of Digital Stomatology, Beijing, PR China

^3^Department of Stomatology, the Second Hospital of Shijiazhuang City, Hebei, PR China.

^†^ These authors contributed equally to this work and shared the first authorship.

* **Correspondence**:

**Dr. Chao Yuan** (corresponding author, chaoyuan@bjmu.edu.cn)

**Ms. Jiahui Niu** (co-corresponding author, [niujiahui99@163.com](mailto:niujiahui99@163.com))

**Prof. Shuguo Zheng** (co-corresponding author, kqzsg86@bjmu.edu.cn)

No.22, Zhongguancun South Avenue, Haidian District, Beijing,100081, PR China

This document includes:

Table S1-S9

Fig. S1-S6

**Results**

**Table S1: Baseline demographic characteristics and periodontal/systemic status of the study participants.**

| Variables | Group A(n=24) | Group B(n=18) | *P*-value |
| --- | --- | --- | --- |
| Age(years) | 68.42±5.69 | 65.06±5.68 | 0.065 |
| Gender(F/M) | 17/7 | 13/5 | 0.921 |
| Teeth | 26.21±2.36 | 24.72±2.65 | 0.063 |
| Duration of diagnosis of T2DM (months) | 152.96±87.91 | 115.78±45.57 | 0.084 |
| Insulin use (Yes/No) | 3/21 | 2/16 | 0.891 |
| BMI(mean kg/m2) | 25.50±3.75 | 24.35±2.88 | 0.287 |
| PD(mm) | 3.22±0.59 | 3.13±0.48 | 0.588 |
| BI | 2.65±1.03 | 2.11±0.63 | 0.041* |
| CAL(mm) | 0.41(0.01-1.36) | 1.07±0.69 | 0.121 |
| HbA1c(%) | 6.85(6.35-8.03) | 6.55(6.30-7.23) | 0.309 |
| FPG(mmol/L) | 7.94±1.91 | 7.41±1.80 | 0.369 |
| CHOL(mmol/L) | 4.81±0.96 | 4.51±1.01 | 0.332 |
| TG(mmol/L) | 1.32(1.08-2.10) | 1.27(0.90-1.92) | 0.703 |
| HDL(mmol/L) | 1.52±0.29 | 1.33±0.24 | 0.039* |
| LDL(mmol/L) | 2.69±0.84 | 2.47±0.72 | 0.398 |
| CRP(mmol/L) | 1.30(0.25-3.59) | 0.25(0.25-0.81) | 0.032* |

Note: Normally distributed data are presented as mean ± SD, whereas non-normally distributed data are presented as median (Q1–Q3). Group A, intervention group; group B, control group; PD, probing depth; BI, bleeding index; CAL, clinical attachment loss; HbA1c, glycated hemoglobin; FPG, fasting plasma glucose; CHOL, total cholesterol; TG, triglycerides; HDL, high-density lipoprotein; LDL, low-density lipoprotein; CRP, C-reactive protein. *P* values: Comparisons of sex and insulin use were performed using the chi-square test. For the remaining variables, between-group comparisons were conducted using the independent-samples t test for normally distributed data and the Kruskal–Wallis test for non-normally distributed data. **P* < 0.05, ***P* < 0.01, ****P* < 0.001.

**Table S2: ANCOVA analyses adjusting for baseline values.**

| Outcome | Time point | Covariate (baseline) | Group effect (F) | *P*-values* | Partial η² |
| --- | --- | --- | --- | --- | --- |
| BI | 3 months | Baseline BI | 27.813 | <0.001* | 0.416 |
|  | 6 months | Baseline BI | 4.102 | 0.05 | 0.095 |
| CRP(mmol/L) | 3 months | Baseline CRP | 6.279 | 0.017* | 0.139 |
|  | 6 months | Baseline CRP | 0.146 | 0.705 | 0.004 |
| HDL(mmol/L) | 3 months | Baseline HDL | 1.539 | 0.222 | 0.038 |
|  | 6 months | Baseline HDL | 0.236 | 0.63 | 0.006 |

Note: Analysis of intervention effects at 3 and 6 months using analysis of covariance (ANCOVA). Group was entered as a fixed factor and baseline values of each outcome were included as covariates. Partial eta squared (η²) indicates effect size. BI, bleeding index; CRP, C-reactive protein; HDL, high-density lipoprotein. **P* < 0.05.

| Variables | Group A(n=24) | | | Group B(n=18) | | | *P*-values^†^ | *P*-values^‡^ |
| --- | --- | --- | --- | --- | --- | --- | --- | --- |
|  | T0 | T1 | *P*-values^*^ | T0 | T1 | *P*-values^*^ |  |  |
| PD（mm） | 3.22±0.59 | 2.260(2.1,2.5) | 0.000^***^ | 3.13±0.48 | 2.941±0.424 | 0.017^*^ | 0.000^†††^ |  |
| Variation | -0.839±0.539 |  |  | -0.187±0.300 |  |  |  | 0.000^‡‡‡^ |
| BI | 2.65±1.03 | 1.280(1.1,1.4) | 0.000^***^ | 2.11±0.63 | 1.929±0.502 | 0.195 | 0.000^†††^ |  |
| Variation | -1.339±0.989 |  |  | -0.179±0.564 |  |  |  | 0.000^‡‡‡^ |
| CAL（mm） | 0.41(0.01，1.36) | 1.06±0.62 | 0.082 | 1.07±0.69 | 0.72±0.77 | 0.189 | 0.119 |  |
| Variation | 0.329±0.887 |  |  | -0.352±1.092 |  |  |  | 0.031^‡^ |

**Table S3: Changes in periodontal clinical parameters from baseline to the 3-month follow-up.**

Note: Normally distributed data are presented as mean ± SD, whereas non-normally distributed data are presented as median (Q1, Q3). Group A, intervention group; group B, control group; PD, probing depth; BI, bleeding index; CAL, clinical attachment loss.

*Statistically significant difference (within-group comparison between baseline and 3 months; paired t test or Wilcoxon signed-rank test, *P* < 0.05).

***Statistically significant difference (within-group comparison between baseline and 3 months; paired t test or Wilcoxon signed-rank test, *P* < 0.001).

†††Statistically significant difference (between-group comparison at 3 months; independent-samples t test or Mann–Whitney U test, P < 0.001).

‡Statistically significant difference (between-group comparison of changes from baseline to 3 months; independent-samples t test or Mann–Whitney U test, *P* < 0.05).

‡‡‡Statistically significant difference (between-group comparison of changes from baseline to 3 months; independent-samples t test or Mann–Whitney U test, *P* < 0.001).

**Table S4: Changes in systemic clinical parameters from baseline to the 3-month follow-up.**

| Variables | Group A(n=24) | | | Group B(n=18) | | | *P*-values^†^ | *P*-values^‡^ |
| --- | --- | --- | --- | --- | --- | --- | --- | --- |
|  | T0 | T1 | *P*-values^*^ | T0 | T1 | *P*-values^*^ |  |  |
| HbA1c(%) | 6.85(6.35-8.03) | 6.600(6.4,7.0) | 0.073 | 6.55(6.30-7.23) | 6.967±1.085 | 0.591 | 0.740 |  |
| Variation | -0.050(-0.725,0.175) |  |  | 0.089±0.688 |  |  |  | 0.126 |
| FPG(mmol/L) | 7.94±1.91 | 6.82±0.94 | 0.009^**^ | 7.41±1.80 | 7.57±1.76 | 0.455 | 0.083 |  |
| Variation | -0.875(-1.558,-0.003) |  |  | 0.166±0.922 |  |  |  | 0.006^‡‡^ |
| CHOL(mmol/L) | 4.81±0.96 | 4.47±0.91 | 0.052 | 4.51±1.01 | 4.55±0.84 | 0.486 | 0.777 |  |
| Variation | -0.200(-0.650,0.170) |  |  | 0.015(-0.118,0.415) |  |  |  | 0.046^‡^ |
| TG(mmol/L) | 1.32(1.08，2.10) | 1.290(1.0,2.5) | 0.637 | 1.27(0.90-1.92) | 1.761±0.562 | 0.017^*^ | 0.387 |  |
| Variation | -0.020(-0.253,0.328) |  |  | 0.330(0.063,0.772) |  |  |  | 0.077 |
| HDL(mmol/L) | 1.52±0.29 | 1.359±0.273 | 0.003^**^ | 1.33±0.24 | 1.280(1.1,1.4) | 0.908 | 0.525 |  |
| Variation | -0.110(-0.177,-0.003) |  |  | 0.005±0.180 |  |  |  | 0.053 |
| LDL(mmol/L) | 2.69±0.84 | 2.320(1.5,2.9) | 0.007^**^ | 2.47±0.72 | 2.305±0.619 | 0.143 | 0.760 |  |
| Variation | -0.430±0.715 |  |  | -0.168±0.465 |  |  |  | 0.185 |
| CRP(mmol/L) | 1.30(0.25，3.59) | 0.570(0.3，1.2) | 0.205 | 0.25(0.25-0.81) | 0.760(0.3,2.5) | 0.017^*^ | 0.371 |  |
| Variation | 0.00(-1.292,0.360) |  |  | 0.270(-0.055,2.203) |  |  |  | 0.017^‡^ |

Note: Normally distributed data are presented as mean ± SD, whereas non-normally distributed data are presented as median (Q1, Q3). Group A, intervention group; group B, control group; HbA1c, glycated hemoglobin; FPG, fasting plasma glucose; CHOL, total cholesterol; TG, triglycerides; HDL, high-density lipoprotein; LDL, low-density lipoprotein; CRP, C-reactive protein.

*Statistically significant difference (within-group comparison between baseline and 3 months; paired t test or Wilcoxon signed-rank test, *P* < 0.05).

**Statistically significant difference (within-group comparison between baseline and 3 months; paired t test or Wilcoxon signed-rank test, *P* < 0.01).

‡Statistically significant difference (between-group comparison of changes from baseline to 3 months; independent-samples t test or Mann–Whitney U test, *P* < 0.05).

‡‡Statistically significant difference (between-group comparison of changes from baseline to 3 months; independent-samples t test or Mann–Whitney U test, *P* < 0.01).

**Table S5: Changes in periodontal clinical parameters from baseline to the 6-month follow-up.**

| Variables | Group A(n=24) | | | Group B(n=18) | | | *P*-values^†^ | *P*-values^‡^ |
| --- | --- | --- | --- | --- | --- | --- | --- | --- |
|  | T0 | T2 | *P*-values^*^ | T0 | T2 | *P*-values^*^ |  |  |
| PD（mm） | 3.22±0.59 | 2.289±0.318 | 0.000^***^ | 3.13±0.48 | 2.67±0.40 | 0.002^**^ | 0.000^†††^ |  |
| Variation | -0.932±0.594 |  |  | -0.46±0.473 |  |  |  | 0.001^‡‡^ |
| BI | 2.65±1.03 | 1.397(1.175，1.841) | 0.000^***^ | 2.11±0.63 | 1.75±0.54 | 0.039^*^ | 0.232 |  |
| Variation | -1.145±1.154 |  |  | -0.36±0.949 |  |  |  | 0.039^‡^ |
| CAL（mm） | 0.41(0.01，1.36) | 0.959±0.675 | 0.262 | 1.07±0.69 | 0.83±0.82 | 0.724 | 0.868 |  |
| Variation | 0.230±0.982 |  |  | -0.24±1.057 |  |  |  | 0.303 |

Note: Normally distributed data are presented as mean ± SD, whereas non-normally distributed data are presented as median (Q1, Q3). Group A, intervention group; group B, control group; PD, probing depth; BI, bleeding index; CAL, clinical attachment loss.

*Statistically significant difference (within-group comparison between baseline and 3 months; paired t test or Wilcoxon signed-rank test, *P* < 0.05).

**Statistically significant difference (within-group comparison between baseline and 3 months; paired t test or Wilcoxon signed-rank test, *P* < 0.01).

***Statistically significant difference (within-group comparison between baseline and 3 months; paired t test or Wilcoxon signed-rank test, *P* < 0.001).

†††Statistically significant difference (between-group comparison at 3 months; independent-samples t test or Mann–Whitney U test, P < 0.001).

‡Statistically significant difference (between-group comparison of changes from baseline to 3 months; independent-samples t test or Mann–Whitney U test, *P* < 0.05).

‡‡Statistically significant difference (between-group comparison of changes from baseline to 3 months; independent-samples t test or Mann–Whitney U test, *P* < 0.01).

**Table S6: Changes in systemic clinical parameters from baseline to the 6-month follow-up.**

| Variables | Group A(n=24) | | | Group B(n=18) | | | *P*-values† | *P*-values^‡^ |
| --- | --- | --- | --- | --- | --- | --- | --- | --- |
|  | T0 | T2 | *P*-values^*^ | T0 | T2 | *P*-values^*^ |  |  |
| HbA1c(%) | 6.85(6.35，8.03) | 6.85(6.425，7.40) | 0.701 | 6.55(6.30，7.23) | 6.55(5.90，6.78) | 0.447 | 0.380 |  |
| Variation | 0.000(-0.275，0.200) |  |  | -0.318±1.400 |  |  |  | 0.888 |
| FPG(mmol/L) | 7.94±1.91 | 6.610±1.181 | 0.004** | 7.41±1.80 | 6.19±0.88 | 0.000^***^ | 0.485 |  |
| Variation | -1.325±2.020 |  |  | -1.221±2.077 |  |  |  | 0.631 |
| CHOL(mmol/L) | 4.81±0.96 | 4.185±0.949 | 0.006** | 4.51±1.01 | 4.24±0.78 | 0.004^**^ | 0.525 |  |
| Variation | -0.629±1.015 |  |  | -0.540(-0.705，-0.200) |  |  |  | 0.770 |
| TG(mmol/L) | 1.32(1.08，2.10) | 1.335(1.033,1.983) | 0.927 | 1.27(0.90-1.92) | 1.55±0.67 | 0.637 | 0.760 |  |
| Variation | -0.010(-0.195,0.298) |  |  | 0.025±1.083 |  |  |  | 0.929 |
| HDL(mmol/L) | 1.52±0.29 | 1.116±0.217 | 0.000^***^ | 1.33±0.24 | 1.06±0.22 | 0.000^***^ | 0.289 |  |
| Variation | -0.399±0.288 |  |  |  |  |  |  | 0.187 |
| LDL(mmol/L) | 2.69±0.84 | 2.423±0.815 | 0.144 | 2.47±0.72 | 2.375(2.030,2.713) | 0.550 | 0.925 |  |
| Variation | -0.262±0.849 |  |  | 0.037±0.997 |  |  |  | 0.404 |
| CRP(mmol/L) | 1.30(0.25，3.59) | 0.250(0.25，0.25) | 0.007^**^ | 0.25(0.25，0.81) | 0.25(0.25，0.25) | 0.017^*^ | 0.928 |  |
| Variation | -0.89(-2.54，0.00) |  |  | 0.00(-0.40，0.00) |  |  |  | 0.034^‡^ |

Note: Normally distributed data are presented as mean ± SD, whereas non-normally distributed data are presented as median (Q1, Q3). Group A, intervention group; group B, control group; HbA1c, glycated hemoglobin; FPG, fasting plasma glucose; CHOL, total cholesterol; TG, triglycerides; HDL, high-density lipoprotein; LDL, low-density lipoprotein; CRP, C-reactive protein.

*Statistically significant difference (within-group comparison between baseline and 3 months; paired t test or Wilcoxon signed-rank test, *P* < 0.05).

**Statistically significant difference (within-group comparison between baseline and 3 months; paired t test or Wilcoxon signed-rank test, *P* < 0.01).

***Statistically significant difference (within-group comparison between baseline and 3 months; paired t test or Wilcoxon signed-rank test, *P* < 0.001).

‡Statistically significant difference (between-group comparison of changes from baseline to 3 months; independent-samples t test or Mann–Whitney U test, *P* < 0.05).

**Table S7: Changes in HbA1c after stratification of the intervention group by baseline glycemic control.**

| Group A | Group with good blood sugar control(n=6) | Group with poor blood sugar control(n=18) | *P*-values‡ |
| --- | --- | --- | --- |
| Change in HbA1c (%)  from baseline to 3 months | 0.267±0.308 | -0.050(-0.725，0.175) | 0.004‡‡ |
| Change in HbA1c (%)  from baseline to 6 months | 0.200±0.506 | 0.000(-0.650，0.125) | 0.378 |

Note: Normally distributed data are presented as mean ± SD, whereas non-normally distributed data are presented as median (Q1, Q3). Group A, intervention group; group B, control group; HbA1c, glycated hemoglobin.

‡‡Statistically significant difference (between-group comparison of changes from baseline; independent-samples t test or Mann–Whitney U test, *P* < 0.01).

**Table S8: Changes in HbA1c after stratification of the control group by baseline glycemic control.**

| Group B | Group with good blood sugar control(n=7) | Group with poor blood sugar control(n=11) | *P*-values‡ |
| --- | --- | --- | --- |
| Change in HbA1c (%)  from baseline to 3 months | 0.200(0.100，0.300) | -0.027±0.767 | 0.179 |
| Change in HbA1c (%)  from baseline to 6 months | 0.257±0.648 | -0.391±0.736 | 0.075 |

Note: Normally distributed data are presented as mean ± SD, whereas non-normally distributed data are presented as median (Q1, Q3). Group A, intervention group; group B, control group; HbA1c, glycated hemoglobin.

**Table S9: Differential salivary metabolites after nonsurgical periodontal therapy with Benjamini–Hochberg false discovery rate (BH-FDR) adjustment (q < 0.25).**

| Group | Timepoint comparison | Metabolite | Direction | Fold change (FC) | log2(FC) | q value (BH-FDR) | -log10(p) |
| --- | --- | --- | --- | --- | --- | --- | --- |
| A | S1 vs S0 | 13-Eicosenoic acid | Up | 3.4408 | 1.7827 | 6.4901E-07 | 6.1878 |
|  | S2 vs S0 | 13-Eicosenoic acid | Up | 2.8665 | 1.5193 | 0.0017121 | 2.7665 |
| B | S1 vs S0 | 13-Eicosenoic acid | Up | 2.4553 | 1.2959 | 0.038724 | 1.412 |
|  | S2 vs S0 | 13-Eicosenoic acid | Up | 2.2666 | 1.1805 | 0.19978 | 0.69944 |

Note: Group A, intervention group; group B, control group; S0, baseline; S1, 3-month follow-up; S2, 6-month follow-up. Fold change (FC) is calculated as follow-up/baseline; log2(FC) is shown accordingly. “Direction” indicates up- or down-regulation at follow-up relative to baseline. *P* values were adjusted across all tested metabolites using the Benjamini–Hochberg procedure, and q values represent BH-FDR–adjusted *P* values. Metabolites with q < 0.25 (and meeting the preset fold-change / *P*-value screening criteria used in the volcano analysis) are reported in this table.

**Supplementary figures**


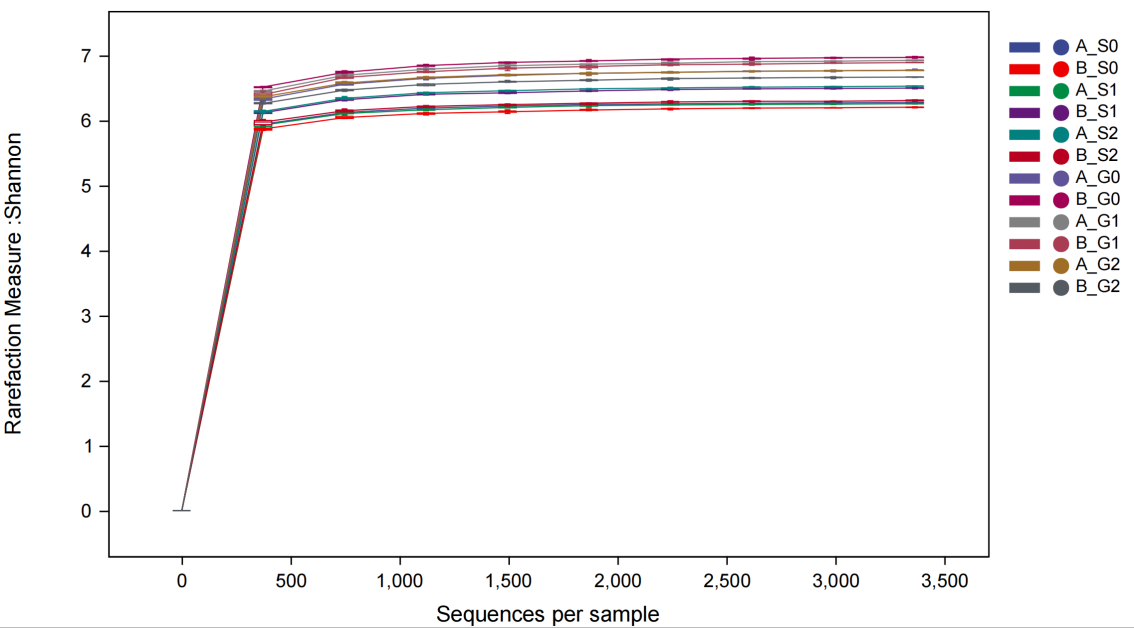


**Figure S1** Rarefaction analysis of the species richness of the salivary and GCF microbiota in each sample.


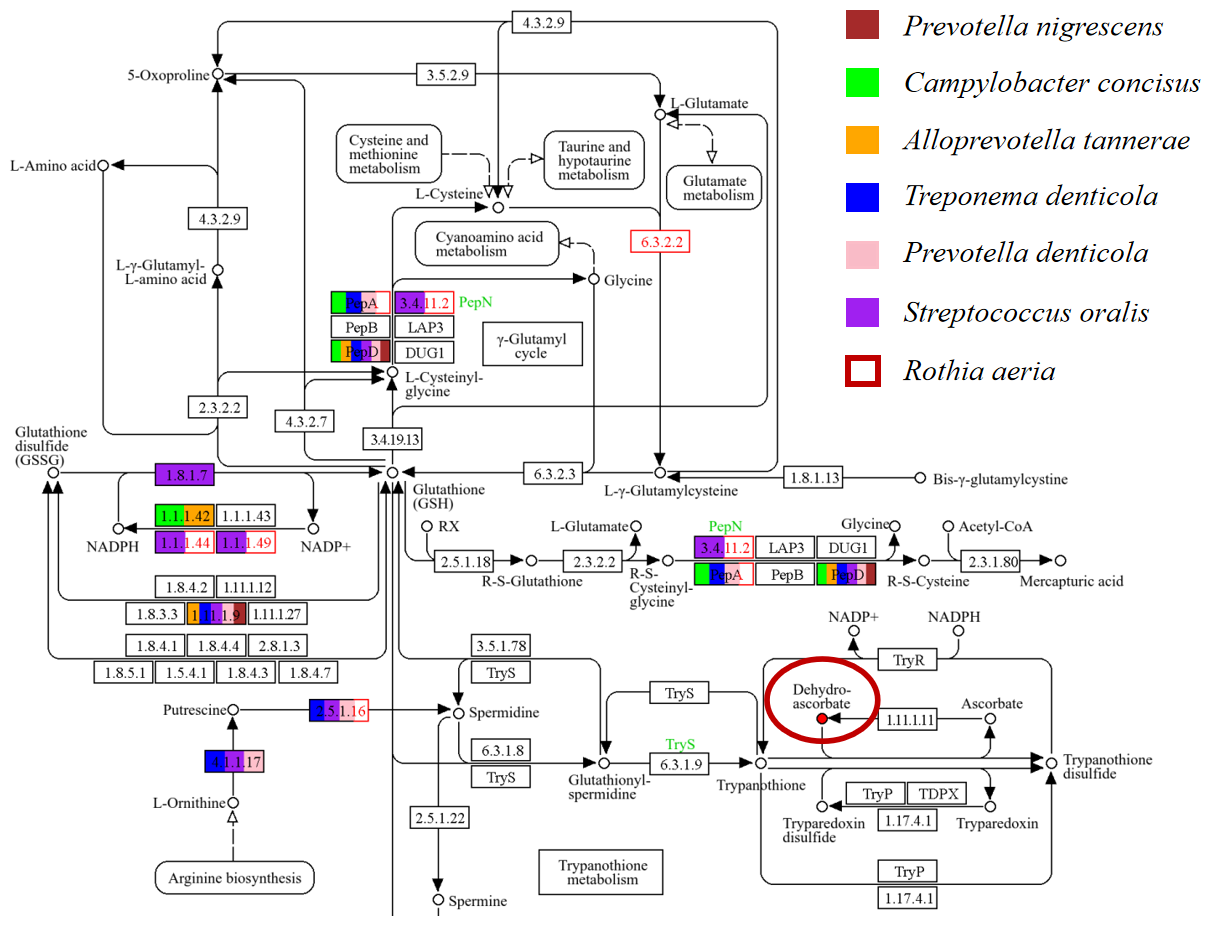


**Figure S2** Enrichment analysis of the glutathione metabolism pathway based on dehydroascorbic acid and differential microbes identified at the 3-month follow-up in the treatment group.


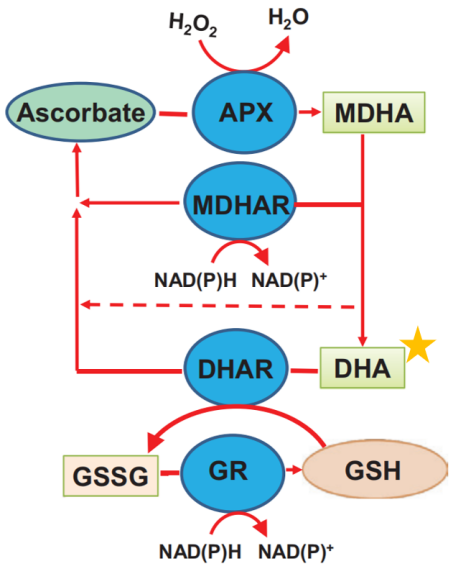


**Figure S3[1]** Schematic illustration of the ascorbate–glutathione cycle (Asada–Halliwell–Foyer cycle) highlighting its role in regenerating the reduced forms of ascorbate and glutathione. APX, ascorbate peroxidase; DHAR, dehydroascorbate reductase; GSH, reduced glutathione; GR, glutathione reductase; GSSG, oxidized glutathione (glutathione disulfide); MDHA, monodehydroascorbate; MDHAR, monodehydroascorbate reductase; DHA, dehydroascorbate.


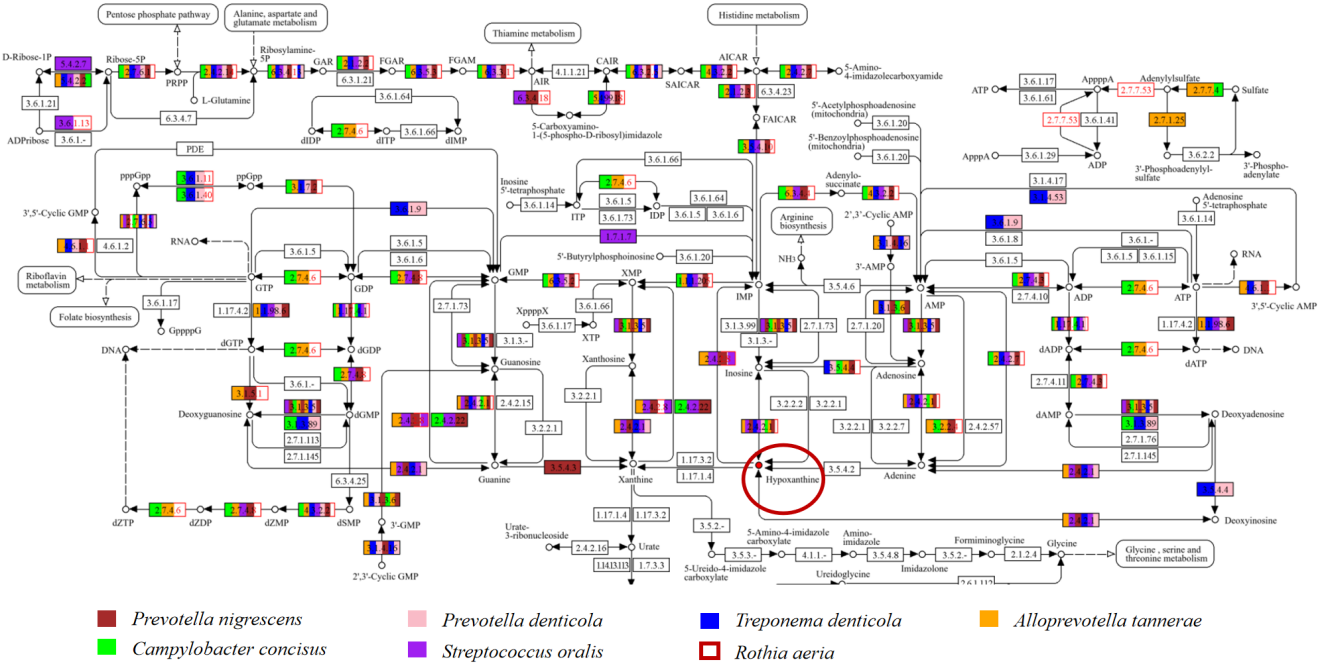


**Figure S4** Enrichment analysis of the purine metabolism pathway based on hypoxanthine and differential microbes identified at the 3-month follow-up in the treatment group.


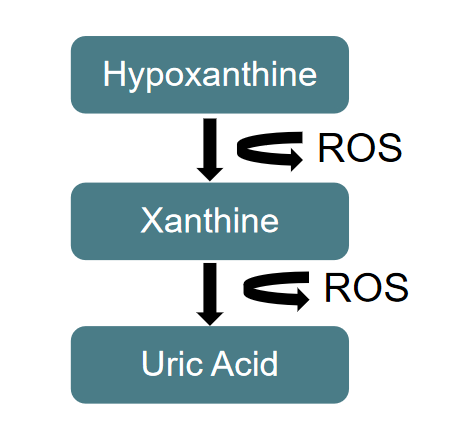


**Figure S5** Schematic of hypoxanthine metabolism within the purine pathway, illustrating its conversion to uric acid and the concomitant generation of reactive oxygen species (ROS)..


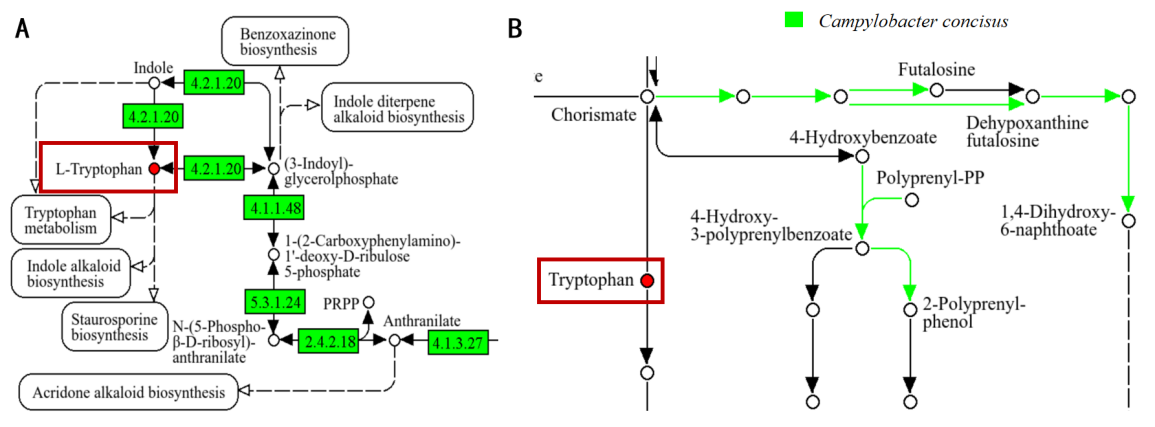


**Figure S6** KEGG pathway annotation integrating *Campylobacter concisus* and tryptophan. (A) Phenylalanine, tyrosine and tryptophan biosynthesis. (B) Biosynthesis of cofactors.

Reference

1. Foyer, C.H. and K. Kunert, *The ascorbate-glutathione cycle coming of age.* J Exp Bot, 2024. **75**(9): p. 2682-2699.
